# Non-invasive detection of early microvascular changes in juveniles with type 1 diabetes

**DOI:** 10.1186/s12933-023-02031-y

**Published:** 2023-10-21

**Authors:** Klaudia Bogusz-Górna, Adriana Polańska, Aleksandra Dańczak-Pazdrowska, Ryszard Żaba, Marta Sumińska, Piotr Fichna, Andrzej Kędzia

**Affiliations:** 1 Department of Pediatric Diabetes, Auxology, and Obesity, Poznan University of Medical Sciences, Poznan, Poland; 2 Department of Dermatology and Venereology, Poznan University of Medical Sciences, Poznan, Poland; 3 Department of Dermatology, Poznan University of Medical Sciences, Poznan, Poland

**Keywords:** Type 1 diabetes, Complications, Microangiopathy, Microcirculation, Capillaroscopy, Photoplethysmography

## Abstract

**Aims/Hypothesis:**

The study aimed to assess the usefulness of capillaroscopy and photoplethysmography in the search for early vascular anomalies in children with type 1 diabetes.

**Methods:**

One hundred sixty children and adolescents aged 6–18, 125 patients with type 1 diabetes, and 35 healthy volunteers were enrolled in the study. We performed a detailed clinical evaluation, anthropometric measurements, nailfold capillaroscopy, and photoplethysmography.

**Results:**

Patients with diabetes had more often abnormal morphology in capillaroscopy (68.60%, *p* = 0.019), enlarged capillaries (32.6%, *p* = 0.006), and more often more over five meandering capillaries (20.90%, *p* = 0.026) compared to healthy controls. Meandering capillaries correlated with higher parameters of nutritional status. In a photoplethysmography, patients with diagnosed neuropathy had a higher percentage of flow disturbance curves (*p* < 0.001) with a reduced frequency of normal curves (*p* = 0.050).

**Conclusions:**

Capillaroscopic and photoplethysmographic examinations are non-invasive, painless, fast, and inexpensive. They are devoid of side effects, and there are no limitations in the frequency of their use and repetition. The usefulness of capillaroscopy and photoplethysmography in the study of microcirculation in diabetic patients indicates the vast application possibilities of these methods in clinical practice.

**Supplementary Information:**

The online version contains supplementary material available at 10.1186/s12933-023-02031-y.

## Introduction

Chronic vascular complications affect vessels of the entire body and are the leading cause of death among diabetic patients [1]. They are reported in up to 26.5% of children and adolescents with disease duration over five years [2]. The search for methods for the early detection of diabetic complications is in progress [3]. The SEARCH for Diabetes in Youth Study found children and adolescents with type 1 diabetes have increased peripheral vascular stiffness compared to healthy controls [4]. Pena et al. studied children and adolescents under 18 with type 1 diabetes without any clinically proven complications. It showed a thickening of the intima-media complex of the carotid artery (cIMT) and increased diameter and twisting of the arterioles in the retinal microvascular bed. Moreover, both changes were correlated [5]. Nussbaum et al. evaluated the sublingual vessels of children with type 1 diabetes without microvascular complications. Compared to healthy controls, patients with diabetes showed a significant reduction of glycocalyx thickness and an increased percentage of vessels with an enlarged diameter [6]. All the abovementioned studies indicate that adequately sensitive research methods allow for identifying vascular disorders among children, even before the onset of clinically evident complications. Moreover, changes are found in different body parts and often correlate. Various methods are applicable for microcirculation disorders, such as laser Doppler flowmetry, orthogonal spectral polarization, near-infrared spectroscopy, optical coherence tomography, or magnetic resonance imaging. However, these methods have limited availability, are sometimes costly, often invasive, time-consuming, and require patient cooperation, making their use in pediatric practice difficult. In contrast to those methods, capillaroscopic and photoplethysmographic examinations are non-invasive, painless, fast, and relatively inexpensive. They are also widely used in clinical practice and relatively easily accessible. We were determined to find a method that met those criteria. Nailfold capillaroscopy is used mainly in connective tissue diseases, especially for differentiating primary and secondary Raynaud's phenomenon and diagnosing systemic scleroderma [7–9]. Also, the abnormalities in the capillaroscopic image reflect systemic vascular disorders in such diseases as acromegaly, arterial hypertension, Crohn's disease, and diabetes [10, 11]. Our study aimed to assess capillaroscopy and photoplethysmography's usefulness in visualizing changes in skin microcirculation in children with type 1 diabetes. Abnormal capillaroscopic images may be present in healthy subjects, including children [12], so we compared diabetic patients with the control group without diabetes—siblings of patients.

## Methods

We recruited children and adolescents with type 1 diabetes from the Department of Pediatric Diabetes, Auxology, and Obesity and the outpatient ambulatory Childhood Diabetes Clinic at Poznan University of Medical Sciences (Poland). The study was designed and realized according to the principles of the Declaration of Helsinki as revised in 2008 and approved by the Local Bioethics Committee of Poznan University of Medical Sciences (185/19). The study group included patients with type 1 diabetes diagnosed according to WHO criteria [13] during insulin therapy for 7.09 ± 3.41 years (min 1 year, max 14.3 years). The enrolled patients ranged from 6 to 18 years of age. The control group included healthy children aged 6–18—siblings of children in the study group. Participants and their parents gave informed consent. We offered participation in our study to all patients under our facility's care, and volunteers were enrolled. Capillary density and morphology change during children’s growth [12]. We intended to search for the effect of diabetes alone in the study group and, as far as possible, eliminate the potential influence of known cofounders. As a result, the control group was formed from the patient’s siblings as they could be genetically and environmentally determined similarly to their brothers and sisters with diabetes. Children with current infection, those with skin lesions in the skin areas subjected to the study, and children with a history of upper limb injury were excluded from the study, as well as individuals presenting the Raynaud phenomenon or diagnosed with autoimmune connective tissue diseases. Treatment with angiotensin-converting enzyme inhibitors (ACEI) or beta-adrenergic blockers also excluded patients from the study [14, 15]. A total of 160 individuals completed the study, 125 of whom had diabetes, and 35 were healthy siblings of diabetic patients.

The research protocol included disease history with additional analysis of medical records, physical examination, capillaroscopy, and photoplethysmography. Anthropometric measurements included height [stadiometr Holstein (United Kingdom)], weight [electronic scales WPT 150, Radwag (Poland)] with the calculation of BMI-SDS [16], bioelectrical impedance analysis [Tanita MC-980 MA (Tanita Corporation, Tokyo, Japan)] defined as body fat mass percentage—BF% and skinfolds thickness [Holtain Skinfold Caliper (United Kingdom)] of the abdominal area, arm above triceps and subscapular region. We performed capillaroscopy and photoplethysmography at the Poznan University of Medical Sciences, Department of Dermatology.

### Capillaroscopy

We examined patients using a Nikon SMZ 800 stereoscopic microscope with digital data archiving. The examiner informed patients about the need to refrain from cosmetic procedures on fingernails for at least 14 days before the examination. We examined the proximal nail fold from the second to fifth fingers of both hands after applying the immersion oil under a 40-fold magnification. We performed capillaroscopy in a room with an ambient temperature ranging from 293,15 to 298,15 K (from 20 to 25 Celcius degrees) after the patient acclimatized for about 20 min. The patient was in a relaxed sitting position with the examined hand resting on the heart’s height. No pressure was applied to the examined finger to prevent vascular flow changes during the examination. The same, highly experienced examiner observed the entire nail bed; all abnormalities were noted. Pictures were taken if abnormalities were found. We based the features assessed in the study on the literature data [15, 17]. Following parameters were assessed: number of capillaries per millimeter, enlarged loops with a diameter > 20 µg), and number of megacapillaries (giant capillaries with loops diameter > 50 µg). A normal range capillary density (≥ 7 capillaries per linear mm), was defined according to Smith et al. [17]. We also looked for avascular fields, hemorrhages, and other morphological abnormalities: meandering, bushy, tortuous, elongated capillaries. According to Smith et al., a bushy or ramified vessel is an abnormal, concave capillary. A tortuous capillary is a vessel when one ascending or descending arm is twisted at least once. Meandering capillaries have marked deviations from the midline with a meandering course but without twisting. We also noted if the patient had an invisible subpapillar venous plexus or granular flow. Granular flow is a slowed flow through vessels with the presence of columns of erythrocytes separated by serous windows. After analyzing the obtained results, we described the children’s summarized results as normal (with no abnormal morphology) or anomalous.

We classified children’s capillaroscopic images into the following groups of the severity [18]:

- 0: No changes in the capillaroscopic image, < 5 capillaries with abnormal morphology.
- 1: Benign changes in the capillaroscopic image, 5–14 capillaries with abnormal morphology, no megacapillaries, avascular fields, or petechiae.
- 2: Moderate changes in the capillaroscopic image, ≥ 15 capillaries with abnormal morphology, no megacapillaries, avascular fields, or petechiae.
- 3: Severe changes in the capillaroscopic image, ≥ 15 capillaries with abnormal morphology, megacapillaries, avascular fields, or petechiae present.

### Photoplethysmography (PPG)

We used a Rheo Screen Compact 2007 photoplethysmograph (Medis, Germany). The examined children comfortably sat at 293,15–298,15 K (20–25 degrees Celsius) ambient room temperature. The measurements were made after room acclimatization for at least 15 min, at the same time of day, between 11:00 and 13:00. During the examination, the person did not speak and avoided moving. Measurements were made sequentially on all fingers, simultaneously on the fingers of the right and left hands. The researcher assessed the shape of each photoplethysmographic curve. We distinguished three curve types: normal curve shape, incorrect curve indicating flow disturbances, and unsuccessful measurement caused by motion artifacts according to the manufacturer's instructions. We assigned measurement as unsuccessful when we found numerous anomalies that the disturbed flow could not explain and probably resulted from the presence of motor artifacts. We summarized the number of normal, abnormal, and unsuccessful curves in each patient.

### Statistical analyses

We performed statistical analyses using IBM SPSS Statistics 23. The Kolmogorov-Smirnow test checked for the normal distribution of quantitative variables. We calculated appropriate descriptive statistics for the analyzed quantitative variables: arithmetic mean with the corresponding standard deviation, minimum and maximum value for the normally distributed data. Median, minimum, and maximum were used to describe non-normally distributed data. For variables showing non-compliance with the normal distribution, we performed statistical analyses using non-parametric tests and parametric tests for the remaining variables. We used Student's t-tests, Mann–Whitney U tests, one-way analyses of variance, Kruskal–Wallis tests, χ2 tests, and Fisher's exact tests to assess the relationship of variables' relationship compare the study groups, depending on the scale used and the normality of the distribution. We also performed correlation analyses with the Pearson r coefficient for quantitative variables with a normal distribution. For the remaining variables, or analyzed dichotomous or ordinal variables, the rank correlation ρ Spearman was used. We complied ordinal variables so that a higher rank value indicated a higher feature level. In the case of dichotomous variables, a higher rank value indicated the feature's presence. The value of *p* = 0.05 was considered the level of significance.

As a result of choosing siblings for the control group, both groups were of incomparable size. Children from the control group were also younger, predominately boys. To make sure such cofounders as age and sex are not influencing our results, we removed some individuals from the study group and divided subjects into three groups. Group A represents all of the patients with diabetes; we used their data for non-control statistical analyses. The procedure of removing subjects from the database was performed by a statistician, who only had access to the encoded database and initially had no information about the essence of the study. He eliminated individuals from the study group to obtain no differences regarding sex and age between study and control groups with minimal losses of data. The groups were compared using T-test. As a result, among patients with diabetes, we formed study group B, compatible with the control group (Group C) regarding sex and age, to perform comparative analyses. We are aware this solution has much potential bias; however, we made sure there is no influence on the choice of subjects.

## Results

### Characteristics of study groups

Characteristics of the studied groups are presented in Table 1.

**Table 1:** Characteristics of studied groups

Characteristics	Group A (n = 125) Mean ± SD min/max	Group B (n = 91) Mean ± SD min/max	Group C (n = 35) Mean ± SD min/max	Comparison: Group B with control
Age (years)	13.32 ± 2.90 min. 6.7–max. 18	12.3 ± 2.64 min. 6.7–max. 16.3	11.15 ± 3.43 min. 6–max. 18	*p* = 0.079
Gender: female (%)	71 (56.80%)	51 (56%)	16 (45%)	*p* = 0.325
Diabetes duration (years)	7.09 ± 3.41 min. 1–max. 14.3	6.66 ± 2.93 min. 2.09–max. 14.2	–	–
BMI-SDS	0.14 ± 0.81 min. (− 1.6)–max. 2.3	0.04 ± 0.73 min. (− 1.6)–max. 1.7	0.14 ± 1.04 min. (− 1.39)–max. 2.5	*p* = 0.590
BF(%)	22.19 ± 5.63 min. 8.4–max. 39.6	21.82 ± 5.17 min. 8.4–max. 33.4	21.36 ± 7.03 min. 10.1–max. 34.6	*p* = 0.785
Skinfold: arm (mm)	14.44 ± 8.3 min. 2–max. 36	15.08 ± 6.46 min. 2–max. 30	11.30 ± 5.42 min. 4–max. 22	*p* = 0.017
Skinfold: abdomen (mm)	15.46 ± 6.65 min. 2–max. 38	13.4 ± 7.91 min. 2–max. 36	10.20 ± 7.36 min. 1—max. 29	*p* = 0.102
Skinfold: back (mm)	12.87 ± 6.84 min. 1–max. 38	11.91 ± 5.78 min. 4–max. 28	9.75 ± 6.90 min. 1–max. 30	*p* = 0.151
DID (U/kg)	0.8 ± 0.17 min. 0.39–max. 1.23	0.79 ± 0.17 min. 0.41–max. 1.19	–	–
HbA_1c_ 3 months Median (mmol/mol)	54 min. 31–max. 216,9	54 min. 40–max. 216,9	–	–
HbA_1c_ 3 months (%) Median	7,10 min. 5–max. 22	7,10 min. 5.8–max. 22	–	–
CSII	95 (76%)	70 (77%)	–	–
Retinopathy	0 (0%)	0 (0%)	–	–
Albuminuria	3 (2.40%)	1 (1.10%)	–	–
Neuropathy	2 (1.60%)	1 (1.10%)	–	–
Hypertension	10 (8%)	5 (5.50%)	–	–
Dyslipidemia	52 (41.60%)	40 (43.95%)	–	–
Autoimmune thyroiditis	32 (25.60%)	26 (28.60%)	0/0%	p < 0.001
Celiac disease	14 (11.20%)	11 (12,10%)	1/2.90%	*p* = 0.177
Asthma	11 (8.80%)	9 (9,90%)	1/2.90%	*p* = 0.281
Epilepsy	0 (0%)	0 (0%)	1/2.90%	*p* = 0.321
Vitiligo	5 (4%)	5 (5,50%)	1/2.90%	*p* = 0.477

*SD* standard deviation, *min.* minimum, *max* maximum, *BMI-SDS* body mass index standard deviation score, *BF (%)*—body fat mass percentage, *HbA1c* glycated hemoglobin the result obtained during last three months, *DID (U/kg)* daily insulin dose in units per kilogram, *CSII* continuous subcutaneous insulin infusion, *p* level of significance

### Nailfold capillaroscopy

Results are shown in Table 2. We didn’t perform capillaroscopy on five children in Group B due to cosmetic procedures or finger injuries preceding the examination.

**Table 2:** Selected vascular anomalies in nailfold capillaroscopy in relation to the group studied

Features of the capillaroscopic image	Group B (n = 86) n/%	Group C (n = 35) n/%	*p*
Enlarged capillaries	28/32.60	4/11.40	0.006
Meandering capillaries	25/29.10	7/20.00	0.152
Bushy capillaries	1/1.20	0/0.00	1
Tortuous capillaries	35/40.70	13/37.20	0.488
Elongated capillaries	10/11.60	3/8.60	0.256
Hemorrhages	8/9.30	2/5.70	0.183
Cap shaped hemorrhages	1/1.20	1/2.90	0.497
Decreased capillary density	1/1.20	2/5.70	0.200
Not visible sub-venous plexus	2/2.30	1/2.90	1
Granular flow	7/8.10	0/0.00	0.191
Abnormal morphology			
Yes	59/68.60	16/45.70	0.019
No	27/31.40	19/53.30	

*p* level of significance, *n* number of studied patients

**Fig. 1:**
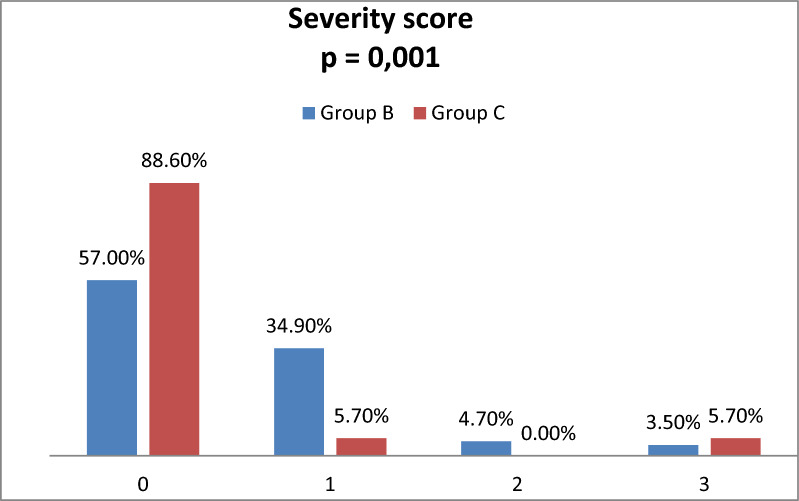
Comparison of group B with Group C in terms of the severity of changes in nailfold capillaroscopy. Severity score: 0: No changes. 1: Benign changes. 2: Moderate changes. 3: Severe changes

In the case of the most common lesions, enlarged, tortuous, bushy, elongated vessels and hemorrhages, we assessed the number of and defined them as single (≤ four abnormalities found) or multiple (≥ five changed vessels or petechiae). Among patients with diabetes, multiple meandering capillaries were significantly more common (*p* = 0.026). We observed a similar trend in the case of enlarged and tortuous vessels, but the relationships were not statistically significant (*p* = 0.063 and *p* = 0.076, respectively). Based on the above observations, researchers assigned the capillaroscopic images to individual groups according to the severity scale used. As shown in Fig. 1, we noted a greater intensity of changes in the group of patients with diabetes. We included detailed results in supplemental data.

**Fig. 2:**
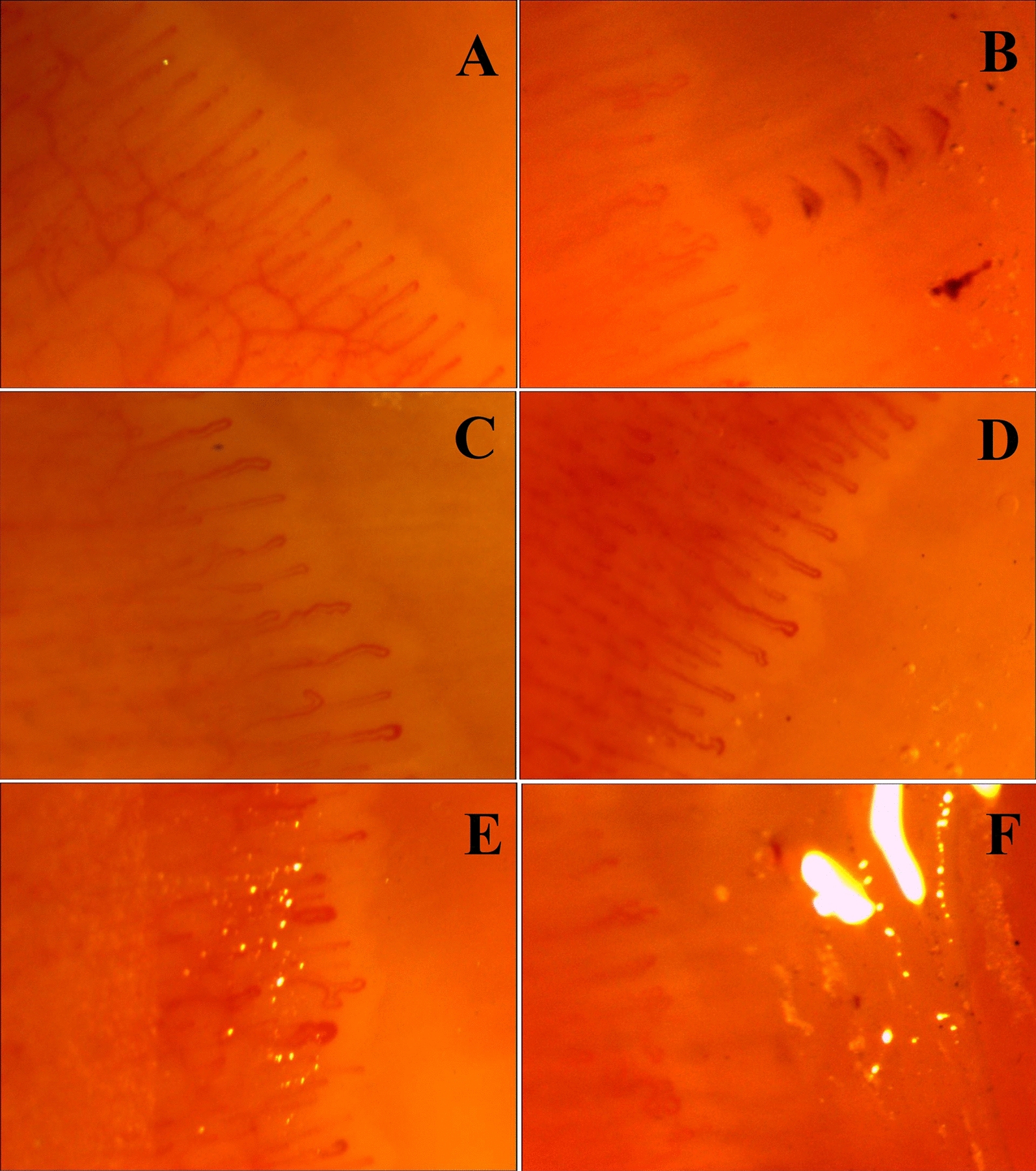
Examples of capillaroscopy findings in analyzed groups of patients. **A** Normal capillaroscopy image, sub-venous plexus visible. **B** Meandering capillaries, cap-shaped hemorrhages **C** Elongated, meandering, and enlarged capillaries. **D** Enlarged and meandering capillaries **E** Enlarged (with cutoff width for megacapillary) and tortuous capillaries. **F** Tortuous capillaries

Examples of capillaroscopy images are shown in Fig. 2.

We performed capillaroscopy on 118 children from Group A. We didn’t examine seven children in Group A due to cosmetic procedures or finger injuries preceding the examination. We have found a positive correlation between parameters of the nutritional status and meandering capillaries and a negative correlation with the presence of granular flow (Table 3). We found a similar relationship in the case of glomerular vessels; however, glomerular vessels were found in capillaroscopy only in one person from the study group. We found no other statistically significant correlations between results and clinical features (including age, duration of diabetes, and parameters of metabolic control). Correlation between the presence of comorbidities and characteristics of the capillaroscopic image was also studied. We’ve found no statistically significant result except one—patients with vitiligo had more often elongated capillaries (*p* = 0.038).

**Table 3:** Correlations between capillaroscopic findings and selected clinical parameters in studied Group A

Features of the capillaroscopic image		Age (years)	BMI SDS	BF (%)	Skinfold arm (mm)	Skinfold abdomen (mm)	Skinfold subscapular (mm)	DID U/kg in sections
Enlarged capillaries	R_S_	0.164	0.007	0.010	0.016	0.027	0.066	− 0.015
	*p*	0.076	0.939	0.914	0.869	0.780	0.253	0.868
Meandering capillaries	R_S_	0.129	0.260	0.215	0.257	0.218	0.295	− 0.029
	*p*	0.162	0.004	0.025	0.007	0.022	0.002	0.756
Tortuous capillaries	R_S_	− 0.120	− 0.082	− 0.124	− 0.177	− 0.092	− 0.130	− 0.061
	*p*	0.195	0.375	0.203	0.065	0.340	0.177	0.510
Elongated capillaries	R_S_	− 0.010	0.139	− 0.173	< 0.001	< 0.001	0.013	0.021
	*p*	0.915	0.132	0.074	0.998	0.998	0.890	0.824
Bushy capillaries	R_S_	-0.020	0.069	− 0.017	0.044	0.056	0.021	0.204
	*p*	0.827	0.456	0.861	0.649	0.562	0.826	0.026
Hemorrhages	R_S_	0.121	0.099	− 0.069	− 0.019	0.038	0.034	0.194
	*p*	0.193	0.288	0.480	0.844	0.691	0.722	0.036
Cap shaped hemorrhages	R_S_	0.091	− 0.028	− 0.187	− 0.145	− 0.105	− 0.100	− t0.044
	*p*	0.329	0.764	0.052	0.131	0.274	0.300	0.640
Granular flow	R_S_	− 0.002	− 0.201	− 0.212	− 0.252	− 0.071	0.102	− 0.004
	*p*	0.983	0.029	0.213	0.008	0.460	0.291	0.968
Abnormal morphology	R_S_	0.004	0.058	− 0.022	− 0.055	-0.038	− 0.010	− 0.048
	*p*	0.966	0.532	0.823	0.567	0.696	0.915	0.605
Severity scale	R_S_	− 0.002	0.105	0.025	0.047	0.064	0.108	− 0.059
	*p*	0.983	0.259	0.795	0.629	0.510	0.262	0.550

*BMI-SDS* body mass index standard deviation score, *BF (%)* body fat mass percentage, *mm* millimeters, *DID* daily insulin dose in units per kilogram expressed in sections (below 0.5 U/kg, between 0.5 U/kg and 1.0 U/kg, or above 1.0 U/kg), *p* level of significance, *R*_*S*_ Spearman’s rank coefficient

### Photoplethysmography results

**Fig. 3:**
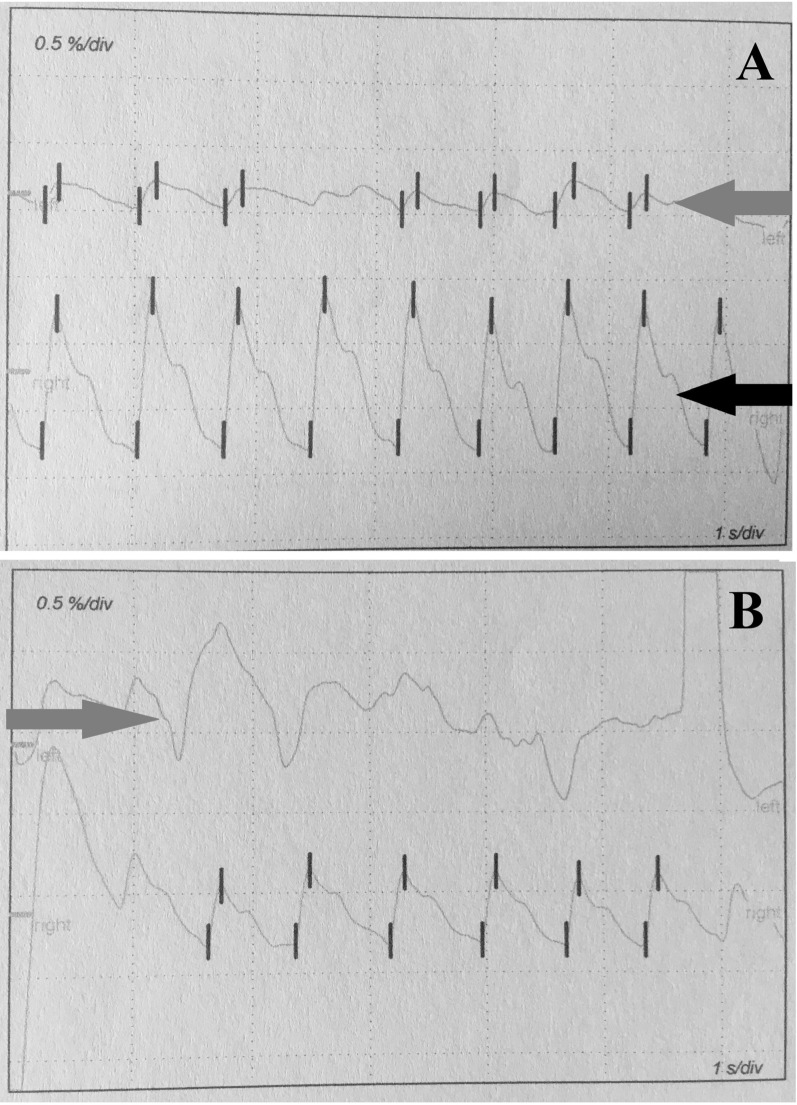
Examples of photoplethysmographic curves**. A** Normal curve shape (black arrow) and a curve ndicating flow disturbances (grey arrow). **B** Unsuccessful measurement (grey arrow)

We performed photoplethysmography on 120 patients from Group A, 87 from Group B, and 34 from the control group. Remaining participants were not tested due to a lack of cooperation. Examples of photoplethysmographic curves are presented in Fig. 3. The groups had no significant difference regarding the number of curves assigned to each category. Detailed results are present in supplemental data.

We assessed the relationship between the mean number of fingers with specific curve shapes and the clinical parameters of diabetic patients. Among the discussed features, adipose tissue content and the number of curves assessed as correct correlated positively. The value of the last measurement of HbA1c positively correlated with the number of normal curves, which means that in patients with higher HbA1c values, we assessed more PPG curves as normal. HbA1c was not associated with the more frequent presence of flow disturbance curves. We included detailed data in the supplemental material. Patients with diagnosed neuropathy had a higher percentage of flow disturbance curves with a reduced frequency of normal curves. Normal curves were found in the mean of 5.00 ± 0.00 fingers of persons with neuropathy and 6.62 ± 3.10 fingers of patients without neuropathy (*p* < 0.001). Patients with neuropathy had incorrect curve shapes on average in 4.00 ± 1.41 fingers, and patients without neuropathy on average in 1.58 ± 1.72 fingers (*p* = 0.050). We observed a similar tendency in patients with albuminuria but without statistical significance; however, it’s important to remember that only a few patients had microangiopathic complications. People with celiac disease had more frequently normal curves (*p* = 0.035), with no association between celiac disease and the presence of curves indicative of flow disturbances.

## Discussion

We attempt to analyze early microcirculation disturbances in a population of children with diabetes. Data on this subject are limited [4–6, 19–22]. We used two independent tools (capillaroscopy and plethysmography) to assess the extent and severity of possible microangiopathic complications related to many variables, including the metabolic status of patients with and mainly without clinically evident microangiopathy. Standardized protocols in interpreting capillaroscopy are commonly used in rheumatic diseases [9]. In our study, we adopted several aspects of this standard. Nevertheless, limiting only to describing morphology as abnormal or normal would not allow us to evaluate other disturbances with potential importance in diabetes. We designed and performed the study according to Smith et al. [17], with modifications that seemed appropriate to our research field. After analyzing the literature on changes in capillaroscopy in children with diabetes, we decided to use similar parameters to maintain, to some extent, the comparability of studies and the possibility of referring to existing data [20–25]. We used an unstandardized method of describing photoplethysmography on the same principle. We intended to find easy to use in clinical practice screening tools. In the literature, plethysmography methodologies used to study microcirculation in patients with diabetes are usually highly complicated with using specific equipment and software to interpret data [26–28]. Such a method would exclude our aim to find a simple tool, so we decided to describe the shape of the photoplethysmographic curve according to the manufacturer's instructions.

Unstandarised methodology in our study might be considered a caveat of the study, although one of our aims was to indicate the need for more investigations in the area of nailbed microcirculation in the course of type 1 diabetes in children. We believe that our research has confirmed this thesis and thus might be considered as a pilot study. Extensive observational studies are needed in the juvenile population to identify whether anomalies in capillaroscopy result from the course of the disease and its complications or are a natural consequence of children’s growth and development. Secondly, longitudinal research for associations between microvascular morphological changes with capillaroscopy and flow changes with photoplethysmography can be performed to assess the predictive role in clinical microvascular complications such as microalbuminuria, neuropathy, and retinopathy. On the other hand, we observe very effective progress in the technical support for diabetes control, increasing the chance of diminishing the risk of complications, which might affect the results of longitudinal observations. Undoubtedly, more numerous and well-adjusted groups of investigated patients should be reinvestigated in cross-sectional studies.

Only five patients in our study group had microangiopathic complications (4.3%)—three patients had albuminuria, and two had neuropathy. In the pediatric population, the clinical manifestation of advanced complications is rare among patients because of the relatively short course of illness. Unfortunately, many patients presented excess body weight (16%), arterial hypertension (8,7%), and dyslipidemia (41,6%), so we may assume microangiopathic complications will develop soon due to risk factors [29, 30]. Early identification of patients at risk of microangiopathic complications is essential due to the potential reversibility or stopping of the progression of changes.

Kuryliszyn et al. found abnormal capillaroscopic images in 81% of adult patients with diabetes. More than half of the patients (51%) had been diagnosed with microvascular complications and showed more severe capillaroscopic changes [31]. Despite the patient's younger age, shorter diabetes duration, and a much lower incidence of complications, we also found a high percentage of children with abnormalities in the capillaroscopic examination (68.60%). They also presented more severe changes than the control group. It may indicate the high sensitivity of capillaroscopy, which enables the detection of microvascular abnormalities before revealing microvascular complications such as albuminuria or retinopathy.

We also assessed capillary morphology. Compared to the control group, enlarged and tortuous vessels were found more often in diabetic patients. The results are consistent with other researchers [20, 23, 32–34]. In earlier mentioned papers by Pena et al. [5] and Nussbaum et al. [6], the presence of meandering and enlarged capillaries was found in the retina and sublingual vessels of diabetic children, despite the lack of clinically proven microangiopathic complications in the studied patients. This finding may suggest the generalization of the process going beyond the vessels of the proximal nail fold and may indicate the usefulness of capillaroscopy as reflecting systemic changes [21]. It also might indicate the necessity for a more detailed description of abnormal capillary morphology in patients with diabetes compared to rheumatic diseases.

We have found no significant relationship between the presence of alterations in capillaroscopic images and the age of the studied patients, duration of diabetes, or metabolic control. On the other hand, meandering capillaries were correlated with higher adipose tissue content and thicker skinfolds. Moreover, patients with higher daily insulin requirements more often had hemorrhages and bushy capillaries. Algenstaedt et al. showed a decrease in the vessel density and enlargement of microcirculation capillaries in hyperglycemic and obese mice [35]. Our findings may indicate a multifactorial etiopathogenesis of microcirculation complications with the possible contribution of insulin resistance and other metabolic disorders related to obesity and lipotoxicity [36].

In the literature, the relationship between capillaroscopic changes and the duration of diabetes, patients’ age, metabolic control, and other studied parameters is ambiguous. Maldonado et al. [23], Hosking et al. [21], and Abdelmaksoud et al. [24] showed a correlation between capillaroscopy results and different clinical parameters, including microvascular complications. We did not confirm the relationship between capillaroscopic abnormalities and the presence of microvascular complications. Fahring et al., Barchetta et al., and Wielicka et al. showed results similar to ours [20, 22, 25].

Another observation made during this research project is the higher prevalence of elongated capillaries among patients with vitiligo. According to the author’s knowledge, literature data on capillaroscopic images in patients with vitiligo mainly concern people suffering from comorbidities and do not indicate the occurrence of changes typical of vitiligo in capillaroscopy [37, 38]. In addition, healthy people may have elongated vessels [12]. Still, this accidental finding might lead to an exciting direction for future research.

Another tool used to assess the microcirculation in this study is photoplethysmography (PPG), which reflects the pulse waves in the arterioles and is widely used to evaluate blood circulation [28, 39, 40]. Research in diabetes considering PPG is promising [41–44]. However, according to the author’s knowledge, the only published work on assessing vascular flow using PPG in children with diabetes is a 1989 study conducted on a group of 42 patients with diabetes. Researchers found a correlation between vascular complications and changes in the photoplethysmographic examination [26]. Buchs and colleagues [27] and Hai-Cheng Wei et al. [28] proved diabetic neuropathy highly correlated with abnormalities in PPG results.

PPG offers many possibilities for various evaluations of the obtained results. We decided to analyze the shape of the curves according to the manufacturer’s instructions. Due to the potential possibility of using the method in clinical practice, the author wanted the developed tool to be easy to use and not require additional specialized software. Rosato et al. also assessed the shape of the curves in a study concerning systemic scleroderma and primary Raynaud’s sign patients [45]. In our study, patients with known peripheral neuropathy had a higher percentage of flow disturbance curves with a reduced frequency of normal shape curves. However, the reader should remember that the number of patients diagnosed with neuropathy in the study group is small. Still, this fact seems significant due to literature reports on the relationship between disorders in photoplethysmography and diabetic neuropathy.

An interesting observation was finding more frequent curves assessed as normal in the group of patients with celiac disease. Celiac disease is a co-morbid disease that may increase the risk of vascular disease. A large cross-sectional study by Rohrer et al., based on the analysis of data from a population of 56,514 patients, identified celiac disease as an independent risk factor for complications such as retinopathy and nephropathy in patients with type 1 diabetes mellitus [46]. Our analysis found no more frequent occurrence of curves showing flow disorders in the group of patients with celiac disease. This link, and perhaps a random statistical finding, requires confirmation.

We noted a high percentage of unsuccessful measurements resulting from the lack of cooperation with the subjects and the presence of motor artifacts. In researching the pediatric population, it is necessary to consider this critical factor. Nevertheless, it seems that the method of assessing photoplethysmographic curves is relatively simple, helpful in examining the microcirculation in diabetic patients, and allows for distinguishing the risk group of patients at risk of developing diabetic neuropathy. Further studies on the more extensive group of patients are needed.

The research protocol’s advantage is including healthy siblings of diabetic children due to the similar environmental and genetic factors in both groups, potentially affecting the presented research results. Unfortunately, the small size of the control group is a consequence. A significant limitation of the project was obtaining data on the metabolic control of patients, such as HbA1c values, comorbidities, microvascular complications, and risk factors for complications based on medical history and available medical documentation. It would be beneficial to conduct up-to-date laboratory analyses and additional tests on the population of the studied children. This project does not consider an extension of diagnostics due to the respondents’ reluctance to increase the number of medical interventions in children with diabetes and their healthy siblings. During the qualification for the study, the author repeatedly met with patients’ objections to any additional medical intervention, including blood withdrawn for laboratory analyses. The respondents and their parents made participation in the project dependent on the non-invasive nature of the procedures undertaken and the lowest possible burden on patients and their siblings. Undoubtedly, several parameters, such as current glycemia level during the examination in both groups or cholesterol levels in the control group, would be beneficial and increase the study’s quality. Unfortunately, we had no possibility to perform those. Nevertheless, the obtained data on clinical parameters are exceptionally complete due to the comprehensive care of patients and regular evaluation and screening as part of standard diabetes care. Moreover, the use of data from medical records and medical history made it possible to obtain information on not only the current HbA1c value but also data on the measurements of glycosylated hemoglobin obtained over the last year, which, due to the influence of chronic metabolic control on the presence of complications, is of particular importance [47–49]. On the other hand, a control group formed from siblings of patients might be considered a limitation of the study. Perhaps abnormalities found in capillaroscopy are a result of genetic factors. All individuals with found anomalies remain under our care and are closely monitored.

We believe our study highlights an important topic and could be considered a pilot study, with the need for more extensive research in the area. Our findings may help design large studies, which might bring the answers we seek. The results we provide may indicate the high sensitivity of capillaroscopy, which enables the detection of microvascular abnormalities before revealing microvascular complications such as albuminuria or retinopathy. The presence of meandering and enlarged capillaries in the nailbed in the retina as well as sublingual vessels [5, 6] indicates a correlation between systemic microcirculation and the condition of the proximal nail fold capillaries. Still, unlike rheumatoid diseases, capillaroscopic patterns typical for diabetic microvascular abnormalities have not been found. However, further research in this area is necessary due to the heterogeneity of the study groups, different research protocols, and possible variability of capillaroscopic images over time in patients with diabetes.

### Supplementary Information

**Additional file 1:Table S1.** Selected pathological findings in nailfold capillaroscopy in relation to the group studied. **Table S2.** Capillaroscopic changes severity concerning the group studied. **Table S3.** Summary of abnormalities in the capillaroscopic images in group A (n=118). **Table S4.** Presentation of capillaroscopic images severity scale in Group A. **Table S5.** Number of fingers with specific photoplethysmographic curves in the groups. **Table S6.** Correlations between the mean number of fingers with specific photoplethysmographic curves and clinical parameters in group A. **Table S7.** Statistical significance of relationships between the mean number of fingers with specific curves and selected microangiopathic complications and comorbidities. **Figure S1.** relation between the number of fingers with normal photoplethysmographic curve and the presence of neuropathy in group A. **Figure S2.** relation between the number of fingers with photoplethysmographic curve indicating flow disturbances and presence of neuropathy in group A.

## Data Availability

The datasets analyzed during the current study are available from the corresponding author upon reasonable request.

## Abbreviations

aIMT: Intima-media complex of the aorta
BF%: Body fat mass percentage
BMI-SDS: Standarised BMI
cIMT: Intima-media complex of the carotid artery
CSII: Continuous subcutaneous insulin infusion
DID: Daily insulin dose
PPG: Photoplethysmography
