# Supplementary material for: Non-invasive detection of early microvascular changes in juveniles with type 1 diabetes
Source: Cardiovasc Diabetol. 2023 Oct 21;22:285. doi: 10.1186/s12933-023-02031-y (PMC10590527; doi:10.1186/s12933-023-02031-y)
Supplement: Supplementary file 1 — Additional file 1:Table S1. Selected pathological findings in nailfold capillaroscopy in relation to the group studied. Table S2. Capillaroscopic changes severity concerning the group studied. Table S3. Summary of abnormalities in the capillaroscopic images in group A (n=118). Table S4. Presentation of capillaroscopic images severity scale in Group A. Table S5. Number of fingers with specific photoplethysmographic curves in the groups. Table S6. Correlations between the mean number of fingers with specific photoplethysmographic curves and clinical parameters in group A. Table S7. Statistical significance of relationships between the mean number of fingers with specific curves and selected microangiopathic complications and comorbidities. Figure S1. relation between the number of fingers with normal photoplethysmographic curve and the presence of neuropathy in group A. Figure S2. relation between the number of fingers with photoplethysmographic curve indicating flow disturbances and presence of neuropathy in group A. [file 12933_2023_2031_MOESM1_ESM.docx]

Table 1. Selected pathological findings in nailfold capillaroscopy in relation to the group studied

| **Features of the capillaroscopic image** |  | **Group B (n = 86)**  **n / %** | **Group C (n = 35)**  **n / %** | ***p*** |
| --- | --- | --- | --- | --- |
| Enlarged capillaries | ≤ 4  ≥ 5 | 15 / 17.40 %  13 / 15.10 % | 2 / 5.70 %  2 / 5.70 % | 0.063 |
| Meandering capillaries | ≤ 4  ≥ 5 | 7 / 8.10 %  18 / 20.90 % | 6 / 17.10 %  1 / 2.90 % | 0.026 |
| Tortuous capillaries | ≤ 4  ≥ 5 | 14 / 16.30 %  21 / 24.40 % | 10 / 28.60 %  3 / 8.60 % | 0.076 |
| Elongated capillaries | ≤ 4  ≥ 5 | 7 / 8.10 %  3 /3.50 % | 2 / 5.70 %  1 / 2.90% | 1 |
| Hemorrhages | ≤ 4  ≥ 5 | 5 / 5.80 %  3 / 3.50 % | 1 / 2.90 %  1 / 2.90% | 0.860 |

*p* – level of significance, n – number of studied patients

Table 2. Capillaroscopic changes severity concerning the group studied

| **Severity scale** | **Group B (n = 86)**  **n / %** | **Group C (n = 35)**  **n / %** | ***p*** |
| --- | --- | --- | --- |
| **0** | 49 / 57.00 % | 31 / 88.60 % | 0.001 |
| **1** | 30 / 34.90 % | 2 / 5.70 % |  |
| **2** | 4 / 4.70 % | 0 / 0.00 % |  |
| **3** | 3 /3.50 % | 2 / 5.70 % |  |

*p* – level of significance, n – number of studied patients

Table 3. Summary of abnormalities in the capillaroscopic images in group A (n=118).

| **Features of the capillaroscopic image** | **Feature present**  **n/%** | **≤ 4**  **n/%** | **≥ 5**  **n/%** |
| --- | --- | --- | --- |
| Enlarged capillaries | 41 / 34.75 % | 22 / 18.64 % | 19 / 16.10 % |
| Megakapillaries | 0 / 0.00 % |  |  |
| Meandering capillaries | 39 / 33.05 % | 10 / 8.47 % | 29 / 24.58 % |
| Bushy capillaries | 1 / 0.85 % |  |  |
| Tortuous capillaries | 45 / 38.14 % | 21 / 17.8 % | 24 / 20.34 % |
| Elongated capillaries | 15 / 12.71 % | 9 / 7.63 % | 6 / 5.08 % |
| Hemorrhages | 13 / 11.01 % | 7 / 5.93 % | 4 / 3.39 % |
| Cap shaped hemorrhages | 2 / 1.69 % |  |  |
| Decreased capillary density | 3 / 2.54 % |  |  |
| Invisible venous plexus | 5 / 4.24 % |  |  |
| Granular flow | 8 / 6.78 % |  |  |
| Abnormal overall pattern | 80 / 67.80 % |  |  |

n – number of studied patients

Table 4. Presentation of capillaroscopic images severity scale in Group A

| **Severity scale** | **Group A (n = 118)**  **n / %** |
| --- | --- |
| **0** | 65 / 55.08 % |
| **1** | 43 / 36.44 % |
| **2** | 5 / 4.24 % |
| **3** | 5 / 4.24 % |

n – number of studied patients

Table 5. Number of fingers with specific photoplethysmographic curves in the groups

| **Number of fingers** | **Group A**  **Mean ± SD** | **Group B**  **Mean ± SD** | **Group C**  **Mean ± SD** | **Comparison of Group B and C** |
| --- | --- | --- | --- | --- |
| **Normal curve** | 6.59 ± 3.08 | 6.49 ± 3.06 | 7.03 ± 3.40 | *p* = 0.403 |
| **Incorrect curve** | 1.61 ± 1.74 | 1.58 ±1.74 | 1.29 ± 1.29 | *p* = 0.398 |

SD- standard deviation; *p* – level of significance;

Table 6. Correlations between the mean number of fingers with specific photoplethysmographic curves and clinical parameters in group A

|  |  | **Curve shape**  **Normal** | **Curve shape**  **Incorrect** |
| --- | --- | --- | --- |
| **BMI-SDS** | R_P_  *p* | 0.141  0,125 | -0.087  0.347 |
| **BF(%)** | R_P_  *p* | 0.190  0.047 | -0.036  0.707 |
| **HbA_1c_ last** | R_P_  *p* | 0.198  0.033 | -0.113  0.227 |
| **HbA_1c_ mean** | R_P_  *p* | 0.149  0.120 | -0.105  0.274 |

BMI-SDS – body mass index standard deviation score; BF (%) - body fat mass percentage; HbA1c last – glycated hemoglobin – the result obtained during last three months; HbA1c mean – glycated hemoglobin, the arithmetic mean of results obtained during last year. *p* – level of significance; R_P_ – Pearson correlation coefficient

Table 7. Statistical significance of relationships between the mean number of fingers with specific curves and selected microangiopathic complications and comorbidities

|  | **Curve shape**  **Normal** | **Curve shape**  **Incorrect** |
| --- | --- | --- |
| **Albuminuria (n = 3)** | *p* = 0.068 | *p* = 0.288 |
| **Neuropathy (n = 2)** | *p* <0.001 | *p* = 0.050 |
| **Hypertension** | *p* = 0.908 | *p* = 0.196 |
| **Dyslipidemia** | *p* = 0.398 | *p* = 0.878 |
| **Autoimmune thyroiditis** | *p* = 0.712 | *p* = 0.838 |
| **Celiac disease** | *p* = 0.035 | *p* = 0.214 |
| **Asthma** | *p* = 0.282 | *p* = 0.345 |
| **Vitiligo** | *p* = 0.763 | *p* = 0.286 |

*p* – level of significance, n – number of studied patients

| 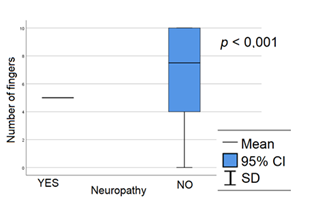  **Fig. 1.** relation between the number of fingers with normal photoplethysmographic curve and the presence of neuropathy in group A | **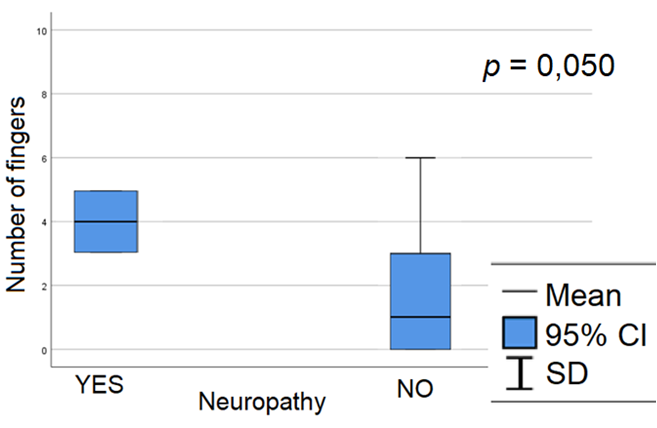**  **Fig. 2.** relation between the number of fingers with photoplethysmographic curve indicating flow disturbances and presence of neuropathy in group A |
| --- | --- |
